# Supplementary material for: Causal Relationship between Aging and Anorexia Nervosa: A White-Matter-Microstructure-Mediated Mendelian Randomization Analysis
Source: Biomedicines. 2024 Aug 16;12(8):1874. doi: 10.3390/biomedicines12081874 (PMC11351342; doi:10.3390/biomedicines12081874)
Supplement: Supplementary file 1 [file biomedicines-12-01874-s001.zip › Supplementary Figure/Figure S4/Singe SNP plot of MR analysis for TL to ubm-b-2017.pdf]

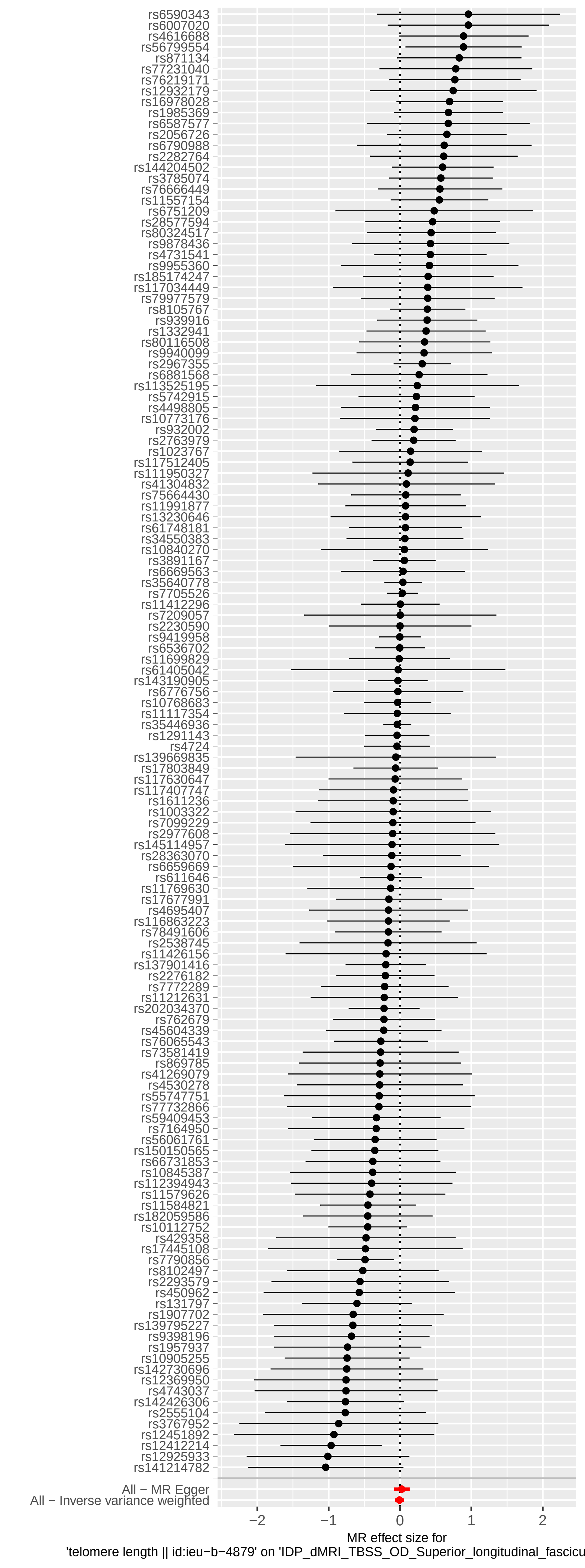

rs6590343
rs6007020
rs4616688
rs56799554
rs871134
rs77231040
rs76219171
rs12932179
rs16978028
rs1985369
rs6587577
rs2056726
rs6790988
rs2282764
rs144204502
rs3785074
rs76666449
rs11557154
rs6751209
rs28577594
rs80324517
rs9878436
rs4731541
rs9955360
rs185174247
rs117034449
rs79977579
rs8105767
rs939916
rs1332941
rs80116508
rs9940099
rs2967355
rs6881568
rs113525195
rs5742915
rs4498805
rs10773176
rs932002
rs2763979
rs1023767
rs117512405
rs111950327
rs41304832
rs75664430
rs11991877
rs13230646
rs61748181
rs34550383
rs10840270
rs3891167
rs6669563
rs35640778
rs7705526
rs11412296
rs7209057
rs2230590
rs9419958
rs6536702
rs11699829
rs61405042
rs143190905
rs6776756
rs10768683
rs11117354
rs35446936
rs1291143
rs4724
rs139669835
rs17803849
rs117630647
rs117407747
rs1611236
rs1003322
rs7099229
rs2977608
rs145114957
rs28363070
rs6659669
rs611646
rs11769630
rs17677991
rs4695407
rs116863223
rs78491606
rs2538745
rs11426156
rs137901416
rs2276182
rs7772289
rs11212631
rs202034370
rs762679
rs45604339
rs76065543
rs73581419
rs869785
rs41269079
rs4530278
rs55747751
rs77732866
rs59409453
rs7164950
rs56061761
rs150150565
rs66731853
rs10845387
rs112394943
rs11579626
rs11584821
rs182059586
rs10112752
rs429358
rs17445108
rs7790856
rs8102497
rs2293579
rs450962
rs131797
rs1907702
rs139795227
rs9398196
rs1957937
rs10905255
rs142730696
rs12369950
rs4743037
rs142426306
rs2555104
rs3767952
rs12451892
rs12412214
rs12925933
rs141214782
All − MR Egger
All − Inverse variance weighted
−2
−1
0
1
2
MR effect size for
'telomere length || id:ieu−b−4879' on 'IDP_dMRI_TBSS_OD_Superior_longitudinal_fascicu
